# Supplementary material for: Experimental warming differentially affects vegetative and reproductive phenology of tundra plants
Source: Nat Commun. 2021 Jun 11;12:3442. doi: 10.1038/s41467-021-23841-2 (PMC8196023; doi:10.1038/s41467-021-23841-2)
Supplement: Supplementary file 1 — Supplementary Information [file 41467_2021_23841_MOESM1_ESM.pdf]

Supplementary Tables

Supplementary Table 1. **Site level climate and experimental warming information**. Average degrees of warming above ambient air temperature achieved in OTCs at each site in our analysis. Weather station information and sources for daily climate data.

Site	OTC Δ°C	Ref	Weather station	Lat	Long	Source
Alexandra Fiord, NU, Canada	1.9	(Bjorkman et al. 2015)[1]	Alexandra fiord field station	78.83	-75.8	Greg Henry (greg.henry [at] ubc.ca)
Endalen, Svalbard	1.8	I Jonsdottir pers. comm	Svalbard Airport/Longyearbyen	78.25	15.47	The Norwegian Meteorological Institute https://seklima.met.no/observations/
Adventdalen, Svalbard	1	(Gillespie et al. 2016)[2]	Svalbard Lufthavn station # SN99840	78.25	15.49	The Norwegian Meteorological Institute https://seklima.met.no/observations/
Utqiaġvik, AK, USA	1.8	(Hollister et al. 2006)[3]	Utqiaġvik field station	71.28	-156.6	Arctic Data Center https://arcticdata.io/catalog/view/doi:10.18739/A2S756K35
Atqasuk, AK,USA	0.9	(Hollister et al. 2006)[3]	Atquasuk field station	70.45	-157.4	Arctic Data Center https://arcticdata.io/catalog/view/doi:10.18739/A2S756K35
Toolik Lake Lake, AK, USA	1.5	(Oberbauer et al. 2007)[4]	Toolik Lake field Station	68.63	-149.6	Arctic Data Center https://arcticdata.io/catalog/view/doi:10.18739/A2Z60C23T
Imnavait Creek, AK, USA	1.6	(Darrouzet-Nardi et al. 2019)[5]	Kuparuk River Watershed (TEON): Imnavait IB	68.61	-149.32	Water and Environmental Research Center http://ine.uaf.edu/werc/werc-projects/teon/current-stations/imnavait/
Latnjajaure, Sweden	1.7	(Molau and Alatato 1998)[6]	Abisko scientific research station	68.33	18.5	Swedish Polar Research Secretariat https://polar.se/en/research-in-abisko/research-data/
Kangerlussuaq, Greenland	1.3	(Pedersen and Post 2008)[7]	Station 4231 Kangerlussuaq/Søndre Strømfjord	67.02	-50.69	Danish Meteorological Institute https://www.isaaffik.org/kangerlussuaq-dmi-meteorological-office
Daring Lake, NT, Canada	0.5	K. Clark, pers. comm	Daring Lake met. station	64.83	-111.63	Karin Clark (Karin_Clark [at] gov.nt.ca)
Healy, AK, USA	1	(Natali et al. 2012)[8]	Eight Mile Lake research watershed	63.88	-149.25	Bonanza Creek LTER https://portal.lternet.edu/nis/metadataviewer?packageid=knb-lter-bnz.453.23
Faroe Islands	0.8	(Fosaa 2015)[9]	Torshavn met station 6011	62.01	-6.78	Danish Meteorological Institute https://www.dmi.dk/publikationer/
Finse, Norway	2.3	(Totland and Alatalo 2002)[10]	Dagali Ii weather station	60.43	8.4	The Norwegian Meteorological Institute https://seklima.met.no/observations/
Jakobshorn, Switzerland	1.2	C. Chisholm, pers. comm	Stillberg meteorological station	46.78	9.87	WSL Institute for Snow and Avalanche Research SLF https://www.envidat.ch/dataset/stillberg-climate
Val Bercla, Switzerland	1	J. Prevéy, pers. comm	Piz Martegnas Schneestation (ENET)	46.58	9.53	WSL Institute for Snow and Avalanche Research SLF
Gavia Pass, Italy	1.7	(Carbognani et al. 2018)[11]	T0065 Careser (Diga)	46.42	10.7	Provincia Autonoma di Trento http://storico.meteotrentino.it/web.htm?ppbm=T0065&rs&1&df
Niwot Ridge, CO, USA	1	(Smith et al. 2012)[12]	Saddle met station	40.05	-105.59	Niwot Ridge LTER https://portal.edirepository.org/nis/mapbrowse?packageid=knb-lter-nwt.405.3
White Mountains, CA, USA	2.1	(Kopp and Cleland 2015)[13]	Barcroft (WMRS) California	37.58	-118.24	Western Regional Data Center https://wrcc.dri.edu/cgi-bin/rawMAIN.pl?nvbarc

Supplementary Table 2**. Subsite (experimental locations within sites) level information on soil moisture, OTC deployment period, and availability of snowmelt data.** Soil moisture levels were assigned to one of three classes: dry, containing roughly < 20% gravimetric water content (GWC); moist 20–60% GWC; or wet > 60% GWC. When possible, sites recorded the first date(s) in each growing season where plots were snow-free to provide information on snowmelt timing in and out of warming chambers.

Site	Subsite	Soil moisture	OTC period	Snowmelt data
Alexandra Fiord, NU, Canada	Cassiope	Moist	Year round	Yes
	Dome Dolomite	Dry	Year round	Yes
	Dome Granite	Dry	Year round	Yes
	Dryas	Moist	Year round	Yes
	Meadow	Wet	Year round	Yes
	Fert	Moist	Year round	Yes
	Saxopp	Dry	Year round	Yes
	Willow	Dry	Year round	Yes
Endalen, Svalbard	Cassiope	Dry	Year round	Yes
	Dryas	Dry	Year round	Yes
	Snowbed	Moist	Year round	Yes
Adventdalen, Svalbard	Heath	Dry	Summer only	Yes
	Meadow	Moist	Summer only	Yes
Utqiaġvik, AK, USA	Dry	Dry	Summer only	Yes
	Wet	Wet	Summer only	Yes
Atqasuk, AK,USA	Dry	Dry	Summer only	Yes
	Wet	Wet	Summer only	Yes
Toolik Lake Lake, AK, USA	Dry	Dry	Summer only	Yes
	Moist	Moist	Summer only	Yes
	Snowfield	Moist	Summer only	Yes
Imnavait Creek, AK, USA	Mat	Moist	Summer only	Yes
Latnjajaure, Sweden	Dry Heath	Dry	Year round	Yes
	Dry Meadow	Dry	Year round	Yes
	Tussock Tundra	Wet	Year round	Yes
	Wet Sedge	Wet	Year round	Yes
Kangerlussuaq, Greenland	Dopey	Moist	Year round	No
	Bashful	Moist	Year round	No
	Doc	Moist	Year round	No
Daring Lake, NT, Canada	Ternplot B	Moist	Summer only	Yes
	Ternplot F	Wet	Summer only	Yes
Healy, AK, USA	Cipher	Moist	Summer only	Yes
Faroe Islands	Dry Meadow	Dry	Year round	No
Finse, Norway	Alpine Moist Heath	Moist	Year round	No
	Stony Snowbed	Moist	Year round	No

	Alpine Meadow	Dry	Year round	No
Jakobshorn, Switzerland	Warmrem	Dry	Summer only	No
Val Bercla, Switzerland	ITEX	Moist	Year round	No
Gavia Pass, Italy	Salix	Moist	Summer only	Yes
	Polytrichastrum	Moist	Summer only	Yes
Niwot Ridge, CO, USA	Sadsnfc	Dry	Summer only	Yes
	Itexnutsnow	Moist	Summer only	No
	Trough	Wet	Summer only	No
	Lefty	Moist	Summer only	No
	Audobon	Moist	Summer only	No
	EK	Dry	Summer only	No
White Mountains, CA, USA	3100	Dry	Year round	No

Supplementary Table 3. **Bayesian hierarchical modeling estimates (mean), error (standard deviation) and 90, 95% Bayesian equal tailed credible intervals [14] of each posterior distribution for the effects of OTC warming on the timing of plant phenology**. Model parameter signifies the slope of either treatment (difference between OTC-CTL, eq. 2) or the interaction of treatment and one of the six spatiotemporal (st) predictors of interest (eq. 3) in days (mean centered i.e. scaled). Bulk Effective sample size (ESS) for each parameter estimate (calculated in brms, Bürkner 2017) reflects the number of independent samples with the same estimation power as the N autocorrelated samples [16]. For Years warming and Site T, the model parameter signifies the difference between OTC and CTL plots per year of warming or degree (°C) in Site T. For Soil moisture, the model parameter signifies the difference between dry sites and moist, wet sites respectively. For OTC period, the model parameter signifies difference between sites with summer-only OTC deployment and year-round deployment. Bolded rows signify models with significant predictors based on Bayesian credible intervals (90% or greater) not crossing zero. For leaf habit, the model parameter signifies the difference between deciduous (D) and evergreen (E) species in response to OTC warming for leaf phenology only.

Model Parameter	Phenophases	Estimate	Error	90% low	95% low	90% high	95% high	Bulk ESS
**OTC**	**Green up**	**-0.054**	**0.035**	**-0.115**	**-0.132**	**-0.002**	**0.008**	**7584**
**OTC**	**Flowering**	**-0.147**	**0.037**	**-0.210**	**-0.225**	**-0.091**	**-0.080**	**9082**
**OTC**	**End of Flowering**	**-0.118**	**0.036**	**-0.175**	**-0.191**	**-0.061**	**-0.047**	**4999**
OTC	Fruiting	-0.160	0.116	-0.346	-0.406	0.006	0.059	4898
**OTC**	**Seed Dispersal**	**-0.139**	**0.067**	**-0.248**	**-0.274**	**-0.034**	**-0.009**	**4261**
**OTC**	**Leaf Senescence**	**0.058**	**0.027**	**0.013**	**0.002**	**0.101**	**0.110**	**10321**
OTC x Years warming	Green up	0.026	0.017	-0.003	-0.007	0.054	0.059	23027
OTC x Years warming	Flowering	0.011	0.012	-0.008	-0.012	0.030	0.034	16498
OTC x Years warming	End of Flowering	0.010	0.016	-0.015	-0.02	0.035	0.041	18699
OTC x Years warming	Fruiting	0.019	0.020	-0.014	-0.020	0.052	0.058	11641
OTC x Years warming	Seed Dispersal	0.034	0.030	-0.016	-0.025	0.081	0.090	8395
OTC x Years warming	Leaf Senescence	0.020	0.019	-0.011	-0.017	0.051	0.058	18266
OTC x Latitude	Green up	-0.009	0.023	-0.046	-0.055	0.030	0.037	6819
OTC x Latitude	Flowering	-0.008	0.024	-0.046	-0.055	0.030	0.040	8069
OTC x Latitude	End of Flowering	-0.023	0.020	-0.056	-0.063	0.010	0.017	14733
OTC x Latitude	Fruiting	0.056	0.086	-0.068	-0.123	0.180	0.229	5823
OTC x Latitude	Seed Dispersal	0.054	0.051	-0.031	-0.053	0.132	0.150	3895
OTC x Latitude	Leaf Senescence	-0.006	0.020	-0.038	-0.044	0.026	0.033	9092
OTC x Soil moist	Green up	-0.041	0.057	-0.133	-0.151	0.054	0.074	5229
**OTC x Soil moist**	**Flowering**	**0.079**	**0.040**	**0.015**	**0.003**	**0.145**	**0.158**	**13549**
OTC x Soil moist	End of Flowering	0.008	0.056	-0.102	-0.124	0.082	0.097	8501
OTC x Soil moist	Fruiting	-0.085	0.100	-0.256	-0.300	0.066	0.098	3796
OTC x Soil moist	Seed Dispersal	-0.039	0.099	-0.196	-0.228	0.128	0.162	5300
OTC x Soil moist	Leaf Senescence	0.035	0.058	-0.060	-0.078	0.130	0.148	6945
OTC x Soil wet	Green up	0.057	0.052	-0.031	-0.053	0.138	0.153	5049
OTC x Soil wet	Flowering	0.054	0.035	-0.002	-0.014	0.111	0.125	12481
OTC x Soil wet	End of Flowering	0.004	0.048	-0.077	-0.102	0.076	0.091	5473
OTC x Soil wet	Fruiting	-0.034	0.082	-0.165	-0.215	0.090	0.130	5590
OTC x Soil wet	Seed Dispersal	0.018	0.082	-0.110	-0.136	0.155	0.189	6710
OTC x Soil wet	Leaf Senescence	0.014	0.059	-0.085	-0.112	0.107	0.128	5475
OTC x OTC Period	Green up	0.115	0.084	-0.012	-0.041	0.259	0.296	3360
**OTC x OTC Period**	**Flowering**	**0.133**	**0.065**	**0.031**	**0.010**	**0.240**	**0.267**	**5538**

OTC x OTC Period	End of Flowering	0.104	0.072	-0.008	-0.032	0.230	0.260	7697
OTC x OTC Period	Fruiting	0.109	0.283	-0.304	-0.445	0.545	0.709	4614
OTC x OTC Period	Seed Dispersal	0.122	0.144	-0.110	-0.170	0.355	0.413	6860
OTC x OTC Period	Leaf Senescence	0.067	0.077	-0.054	-0.080	0.194	0.227	6430
OTC x Site T	Green up	-0.032	0.027	-0.075	-0.084	0.014	0.024	5812
OTC x Site T	Flowering	-0.008	0.024	-0.047	-0.055	0.031	0.038	8760
OTC x Site T	End of Flowering	0.027	0.024	-0.013	-0.022	0.066	0.073	9348
OTC x Site T	Fruiting	0.022	0.056	-0.069	-0.087	0.115	0.136	8435
**OTC x Site T**	**Seed Dispersal**	**-0.093**	**0.056**	**-0.190**	**-0.210**	**-0.007**	**0.007**	**6611**
OTC x Site T	Leaf Senescence	0.004	0.025	-0.036	-0.044	0.044	0.054	10768
OTC x Site-year ΔT	Green up	-0.010	0.020	-0.042	-0.048	0.022	0.028	9247
OTC x Site-year ΔT	Flowering	-0.012	0.012	-0.032	-0.036	0.009	0.013	15236
OTC x Site-year ΔT	End of Flowering	-0.007	0.015	-0.031	-0.035	0.017	0.021	14736
OTC x Site-year ΔT	Fruiting	-0.013	0.020	-0.046	-0.052	0.020	0.026	14237
OTC x Site-year ΔT	Seed Dispersal	0.002	0.024	-0.037	-0.044	0.041	0.049	13118
OTC x Site-year ΔT	Leaf Senescence	0.000	0.019	-0.032	-0.038	0.031	0.037	14292
OTC x Leaf Habit (E)	Green up	-0.035	0.052	-0.121	-0.141	0.049	0.066	1532
OTC x Leaf Habit (E)	Leaf Senescence	-0.037	0.051	-0.121	-0.142	0.048	0.061	3745

Supplementary Table 4. **Group level variation (standard deviation) in days (mean centered, i.e. scaled) of Bayesian hierarchical models for the random effects of treatment grouped by a) species b) site c) site:subsite d) site:year from treatment only models (eq. 2)** for each phenophase. Parameter refers to group level variation (sd) in timing of phenology for each phenophase at the intercept (sd(Intercept)) and slope (sd(treatment OTC)) levels and their correlation cor(Intercept,slope). A positive correlation suggests that groups with larger intercepts also have larger slopes, and vice-versa.

Parameter	Phenophase	Site	Subsite	Site-Year	Species
sd(Intercept)	Green up	0.994	0.589	0.457	0.348
sd(treatmentOTC)	Green up	0.051	0.041	0.017	0.017
cor(Intercept,treatmentOTC)	Green up	0.058	0.521	0.040	0.120
sd(Intercept)	Flowering	0.854	0.502	0.427	0.554
sd(treatmentOTC)	Flowering	0.104	0.031	0.013	0.042
cor(Intercept,treatmentOTC)	Flowering	-0.116	0.264	-0.180	-0.550
sd(Intercept)	End of Flowering	0.590	0.596	0.418	0.715
sd(treatmentOTC)	End of Flowering	0.074	0.028	0.014	0.019
cor(Intercept,treatmentOTC)	End of Flowering	-0.103	-0.076	0.147	-0.122
sd(Intercept)	Fruiting	1.821	0.296	0.461	0.628
sd(treatmentOTC)	Fruiting	0.226	0.043	0.027	0.028
cor(Intercept,treatmentOTC)	Fruiting	-0.278	-0.269	-0.463	-0.256
sd(Intercept)	Seed Dispersal	0.984	0.483	0.465	0.494
sd(treatmentOTC)	Seed Dispersal	0.157	0.045	0.025	0.032
cor(Intercept,treatmentOTC)	Seed Dispersal	-0.608	-0.039	-0.163	-0.002
sd(Intercept)	Leaf Senescence	0.973	0.562	0.419	0.169
sd(treatmentOTC)	Leaf Senescence	0.030	0.027	0.021	0.028
cor(Intercept,treatmentOTC)	Leaf Senescence	-0.079	-0.092	-0.343	0.058

Supplementary Table 5. **Bayesian hierarchical model intercepts and 90, 95% Bayesian equal tailed credible intervals for the treatment only models on 6 plant phenophases**. Model intercepts designate the mean DOY when an event occurred across all sites, species and years. For leaf greenup and senescence estimate the average DOY of leaf greenup was 166 ± 5 days and the average DOY of leaf senescence was 216 ± 5 days. Thus the average species' growing season length across all sites can be estimated as 50 ± 10 days. Model intercepts for flowering and end of flowering estimate the average DOY of flowering across all sites was 181 ± 4 days and the average DOY of end of flowering across all sites was 199 ± 4 days. Thus the average species' flowering season length across all sites can be estimated as 18 ± 8 days. Fruiting estimates were not significantly different from zero (Table S2), thus we cannot accurately estimate the length of the fruiting period.

Model Parameter	Phenophases	Estimate (DOY)	Error	90% low	95% low	90% high	95% high
Intercept	Green up	165.69	4.76	157.92	173.31	156.16	175.11
Intercept	Flowering	181.15	4.23	174.26	188.03	172.71	189.56
Intercept	End of Flowering	199.21	3.89	192.94	205.70	191.67	207.09
Intercept	Fruiting	166.65	11.17	149.00	183.95	143.53	189.23
Intercept	Seed Dispersal	217.09	7.97	204.11	229.82	200.67	232.54
Intercept	Leaf Senescence	216.46	4.8	208.68	224.14	207.02	225.96

Supplementary Table 6. **Group level estimates of Bayesian hierarchical models for the random effects of treatment (OTC-CTL) grouped by a) site b) species c) site:subsite and d) site:year from treatment only models (eq. 2) for each phenophase**. Estimates, standard errors and 95% Confidence intervals based on quantiles (brms default summary) are shown in days. Number of observations (raw data) included in each group level estimate is shown.

a) https://github.com/cour10eygrace/OTC_synthesis_analyses/blob/master/Supplemental_materials/Table6a.csv

Site	Estimate_days	Error_days	CI_lower_days	CI_upper_days	Phenology	n_obs
Adventdalen	-0.85301	0.82332	-2.87067	0.532611	Green up	276
Adventdalen	-2.54435	1.271237	-5.16185	-0.06328	Flowering	279
Adventdalen	-1.7861	1.052365	-3.90673	0.510537	End of Flowering	206
Adventdalen	-3.63991	2.203037	-8.25467	0.557609	Seed Dispersal	208
Adventdalen	0.702899	0.630702	-0.7176	1.866726	Leaf Senescence	107
Alexandra Fiord	-0.88161	0.544204	-2.04236	0.089628	Green up	7407
Alexandra Fiord	-2.11927	0.541129	-3.1802	-1.04147	Flowering	4816
Alexandra Fiord	-2.33054	0.525637	-3.40741	-1.34624	End of Flowering	3550
Alexandra Fiord	-2.77485	0.739449	-4.21734	-1.30538	Fruiting	3678
Alexandra Fiord	-2.7619	1.343319	-5.41195	-0.15038	Seed Dispersal	1047
Alexandra Fiord	0.709495	0.47023	-0.31851	1.593478	Leaf Senescence	3204
Atqasuk	-0.33325	0.527344	-1.29579	0.805455	Green up	7201
Atqasuk	-0.99036	0.579027	-2.20533	0.108	Flowering	5713
Atqasuk	-1.11918	0.573163	-2.27096	-0.04364	End of Flowering	5882
Atqasuk	-0.48348	0.882885	-2.54752	1.042857	Fruiting	4711
Atqasuk	-0.89618	1.096458	-3.18904	1.124238	Seed Dispersal	2799
Atqasuk	0.73378	0.385082	-0.06209	1.472315	Leaf Senescence	4787
Daring Lake	-0.15056	0.996104	-2.11522	1.781178	Flowering	1831
Daring Lake	-0.98395	0.993598	-2.65315	1.172694	End of Flowering	1088
Daring Lake	-0.28376	1.533516	-3.24414	2.777642	Fruiting	880
Daring Lake	-0.52427	1.962999	-4.1313	3.631468	Seed Dispersal	436
Endalen	-3.47429	1.107505	-5.78839	-1.48049	Flowering	344
Endalen	-2.08877	1.192365	-4.77895	0.130682	End of Flowering	117
Endalen	-3.06825	2.222604	-7.82157	1.013353	Seed Dispersal	139
Faroe Islands	-3.14831	1.913316	-7.48036	0.143661	Flowering	58
Faroe Islands	-2.04833	1.236983	-4.86231	0.421048	End of Flowering	58
Finse	-2.69082	1.411972	-5.61817	0.085401	Flowering	200
Finse	-1.68736	1.316488	-4.17327	1.256366	End of Flowering	123
Gavia Pass	-2.10274	0.999668	-4.02926	-0.09939	Flowering	1996
Gavia Pass	-5.81321	1.619977	-8.89527	-2.47031	Fruiting	1863
Gavia Pass	-7.44596	1.937976	-11.147	-3.42051	Seed Dispersal	1796
Healy	-0.98863	0.925938	-3.30477	0.446858	Green up	493
Healy	0.722039	0.528461	-0.43521	1.746771	Leaf Senescence	668
Imnavait	-0.7354	0.707355	-2.31811	0.590293	Green up	816
Imnavait	0.880765	0.555039	-0.14405	2.164804	Leaf Senescence	519
Jakobshorn	-0.66775	0.944213	-2.7125	1.203394	Green up	26
Jakobshorn	-3.52759	1.430489	-6.64752	-1.06912	Flowering	168
Jakobshorn	-1.5568	1.28564	-3.88558	1.432736	End of Flowering	88
Jakobshorn	0.808979	0.614763	-0.41086	2.098712	Leaf Senescence	30
Kangerlussuaq	-1.02286	0.936569	-3.39366	0.382206	Green up	96
Kangerlussuaq	-2.83202	1.234161	-5.40236	-0.49501	Flowering	136
Kangerlussuaq	-3.87475	2.77979	-9.68801	1.123415	Fruiting	62

Latnjajaure	-0.82514	0.93379	-3.01465	0.77269	Green up	50
Latnjajaure	-4.56471	1.333259	-7.29888	-2.13652	Flowering	239
Latnjajaure	-3.0439	1.475333	-6.72505	-0.96252	End of Flowering	87
Latnjajaure	-5.31673	1.990943	-9.5269	-1.75763	Seed Dispersal	118
Latnjajaure	0.679184	0.579318	-0.68641	1.693293	Leaf Senescence	100
Niwot Ridge	-0.60354	0.842962	-2.37045	1.20751	Green up	311
Niwot Ridge	-1.07054	1.05012	-2.95314	1.104861	Flowering	635
Niwot Ridge	-1.29087	1.022345	-2.99181	1.054326	End of Flowering	593
Niwot Ridge	0.735505	0.545353	-0.46651	1.79187	Leaf Senescence	406
Toolik Lake	-0.72582	0.554983	-1.92879	0.281344	Green up	865
Toolik Lake	-2.2531	0.65471	-3.5529	-0.99255	Flowering	933
Toolik Lake	-2.19383	0.730851	-3.72325	-0.84193	End of Flowering	602
Toolik Lake	-1.04511	1.473912	-3.95332	1.81857	Seed Dispersal	347
Toolik Lake	0.875264	0.447587	0.01229	1.820534	Leaf Senescence	1083
Utqiagvik	-0.37538	0.455513	-1.27034	0.539824	Green up	12781
Utqiagvik	-2.45276	0.466009	-3.38878	-1.52635	Flowering	12487
Utqiagvik	-2.28911	0.454365	-3.19332	-1.39203	End of Flowering	9656
Utqiagvik	-2.27729	0.729829	-3.76665	-0.8676	Fruiting	6080
Utqiagvik	-1.6724	1.303144	-4.26759	0.837882	Seed Dispersal	1402
Utqiagvik	0.807163	0.374753	0.065215	1.547225	Leaf Senescence	6134
Val Bercla	-2.48381	1.503281	-5.64742	0.459433	Flowering	158
Val Bercla	-2.00142	1.2757	-4.76375	0.602631	End of Flowering	127
White Mts	-2.60021	1.976551	-6.87302	1.105788	Flowering	18

b) https://github.com/cour10eygrace/OTC_synthesis_analyses/blob/master/Supplemental_materials/Table6b.csv
c) https://github.com/cour10eygrace/OTC_synthesis_analyses/blob/master/Supplemental_materials/Table6c.csv
d) https://github.com/cour10eygrace/OTC_synthesis_analyses/blob/master/Supplemental_materials/Table6d.csv

Supplementary Table 7**. Influence of OTC deployment on snowmelt date in year-round warming sites.** To determine potential effects of OTCs on snowmelt date, we used Bayesian hierarchical modeling with default (non-informative) priors in the R package *brms* for sites/subsites with year-round OTCs and recorded snowmelt information (3 sites, 15 subsites and 29 site-year combinations, n=2246 observations). Model response was the observed DOY that plots were snow free, with a fixed predictor of treatment (OTC, CTL) and random intercepts of site, site:subsite, and site:year. Model estimates show that snowmelt was an average of 1.02 +/- 0.17 days (95% CI= 0.68, 1.36) days earlier in OTCs than control plots for sites with OTCs deployed year-round.

**Model Parameter**	**Estimate**	**Error**	**95% high**	**95% low**	**Bulk ESS**
**Intercept**	**160.12**	**4.51**	**151.05**	**169.13**	**1518**
**OTC**	**-1.02**	**0.17**	**-1.36**	**-0.68**	**2379**

Supplementary Figures

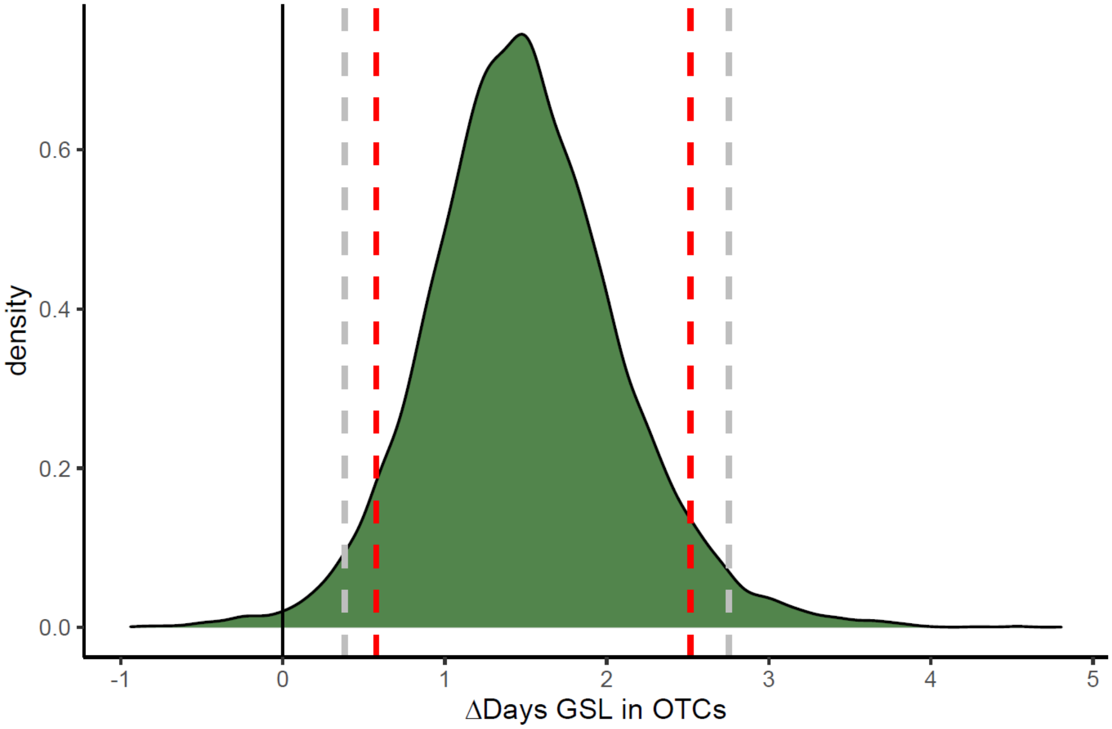

Supplementary Fig 1. **Estimated total shift in species' growing season length.** Differential shifts between greenup and leaf senescence lead to a 1.5-day increase in species' growing season lengths (GSL) under experimental warming. Density plot of the difference in modeled estimates of Green up and Leaf Senescence in response to OTCs. Estimates (in days) are shown on the x-axis and black vertical line denotes zero difference (no change) in the growing season length, while red, grey dashed lines denote the 90, 95% Bayesian credible intervals of estimates. Peaks to the right of the black line indicates a lengthening of the growing season and vice-versa. Created using the ggplot2 package (v 3.3.2)[17] in R.

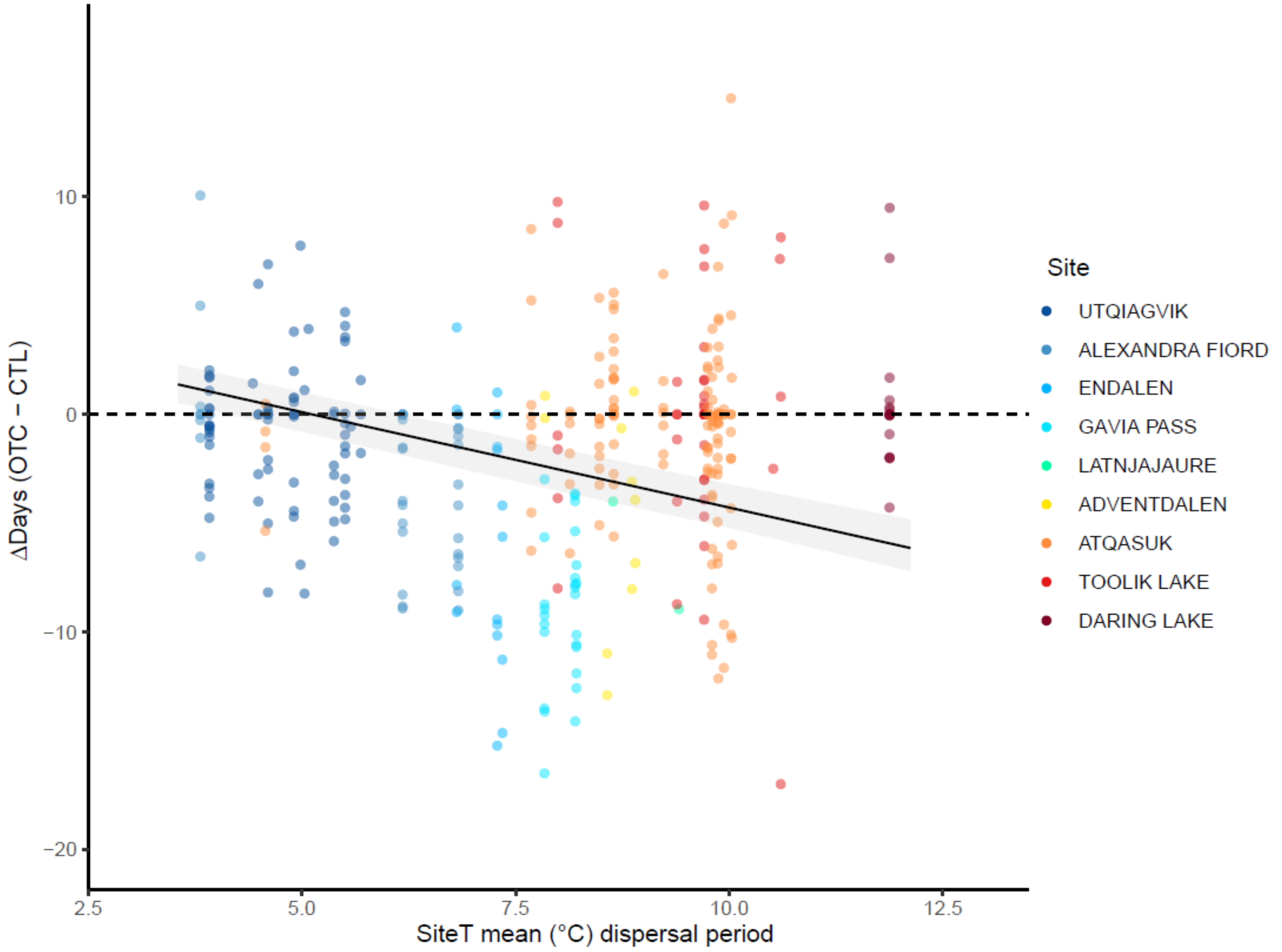

Supplementary Fig 2. **Interaction between site mean temperature and experimental warming on plant phenology**. Seed dispersal in OTCs was earlier for species with warmer climate windows during their dispersal period at a given site. Points reflect the difference (in days) in the timing of seed dispersal for plants growing in OTC versus CTL plots for each replicate (subsite x year x species, n=346) as shown on the y-axis. Black dotted line denotes zero difference (no change) in the timing of phenology between OTC and CTL treatments, while points above or below these lines suggest a delay, or advancement, respectively. The x-axis shows the average site temperature (°C) during the climate window calculated from average seed dispersal dates for each species at a given site across all measurement years (See Methods-Climate data). Color of points designates the site of each replicate and the average site temperature (°C) for all species and measurement years at that site from coldest (blue) to warmest (red). Black solid line shows the posterior fits from the conditional means of the linear predictor site T from our full climate model (Supplementary Methods 1 eq. 3, Table 1) at five simulated values of site T (3.4, 5.6, 7.8, 10.0, 12.2°C) with group level effects held constant and the 5th and 95th quantiles around these fitted values (lower, upper) shown in grey. Created using the ggplot2 package (v 3.3.2)[17] in R.

Supplementary Methods

Supplementary Methods 1. **Bayesian Hierarchical modeling for phenology analyses.**

We used a two-step hierarchical modeling approach to test the effects of OTC warming on plant phenology. First, for each phenophase, we estimated the mean DOY and variation in each treatment (OTC or control) x species x subsite x year combination (hereafter replicate, r). We used interval-censored regression to estimate the mean DOYs (μ_r) and its standard error ($\sigma_{\mu r}$) by fitting an intercept-only model to the data for each replicate (eq.1). This model assumes the (unobserved) DOY on which each phenophase occurred for each individual observation is normally distributed around the replicate mean μ_r with variance σ_r^2 and is estimated using the observed DOYs, censored by the prior visit (DOY) in the survreg function of the survival package in R [18]. Second, we used Bayesian hierarchical modeling with default (non-informative) priors in the R package brms [15] to estimate the effects of OTC warming on plant phenology across sites, species, and years (eq. 2). The response is the previously estimated replicate level mean DOY (μ_r) and associated standard errors ($\sigma_{\mu r}$) which are incorporated into a joint response variable using the resp_se function in brms. Treatment (Trt_r) is an indicator variable for replicates measured in OTCs (1) or control (0), with random effects grouped by species (s), site (k), year within site (y), and subsite within site (j). We used default brms (i.e. non-informative) uniform flat priors for the global intercept (α) and slope (β), and half student_t priors with 3 degrees of freedom and scale = 10 for the variance components σ_r and all group level variances $\sigma_\alpha$and σ_β. For each group (species, site, year within site, and subsite within site), the group level coefficients ($\alpha_{\text{group}}, \beta_{\text{group}}$) , and their correlation $[\alpha_{\text{group}}, \beta_{\text{group}}]$ were modeled using a multivariate normal distribution with means of zero and standard deviation S_{group}, which is the variance-covariance matrix of each varying intercept and slope and their correlation ρ_{group}. Finally, R_{group} is the correlation matrix for the parameter ρ_{group} which has an LKJ-correlation prior distribution with a ζ parameter = 1, which constrains the correlation term uniformly between −1 and 1. Lastly for questions about variation in treatment effects over space, time, and ambient climate, we used the same model structure but with an additional fixed interaction term between treatment and one of six spatiotemporal (St) predictors of interest: 1) Years of warming (continuous, replicate level-r), 2) latitude (continuous, site level-k), 3) water availability (categorical (dry/moist/wet) i.e. <20%, 20-60%, >60% gravimetric water content (GWC), site:subsite level-j), 4) OTC deployment period (categorical (year round/summer only), site level-k), 5) site mean temperature (continuous, site level-k) and 6) site-year temperature anomaly (continuous, site:year level-y) (eq. 3). We included both climate predictors in the same model using a within-group centering approach (described in van de Pol & Wright, 2009) with two interaction terms to distinguish spatial (site- μT) and temporal (siteyear-ΔT) influences of temperature on OTC warming (See Climate Data).

Intercept only models:

$$Y_i \sim \text{Normal}(\mu_r, \sigma_r^2) \qquad \text{eq. 1}$$

Treatment only models:

$$\mu_r \sim \text{Normal}\big(\alpha + \alpha_{s[r]} + \alpha_{k[r]} + \alpha_{y[r]} + \alpha_{j[r]} + (\beta + \beta_{s[r]} + \beta_{k[r]} + \beta_{y[r]} + \beta_{j[r]}) * Trt_r,\ \sigma_r + \sigma\mu_r\big) \qquad \text{eq. 2}$$

$\alpha \sim \text{uniform}\ (-\infty, \infty)$

$\beta \sim \text{uniform}\ (-\infty, \infty)$

$\sigma_r \sim \text{student\_t}(3,0,10)$

$\sigma_{\alpha s} = \text{student\_t}(3,0,10)$

$\sigma_{\beta s} = \text{student\_t}(3,0,10)$

$\sigma_{\alpha k} = \text{student\_t}(3,0,10)$

$\sigma_{\beta k} = \text{student\_t}(3,0,10)$

$\sigma_{\alpha y} = \text{student\_t}(3,0,10)$

$\sigma_{\beta y} = \text{student\_t}(3,0,10)$

$\sigma_{\alpha j} = \text{student\_t}(3,0,10)$

$\sigma_{\beta j} = \text{student\_t}(3,0,10)$

$\begin{bmatrix}\alpha_s\\ \beta_s\end{bmatrix} \sim \text{MVNormal}(\begin{bmatrix}0\\0\end{bmatrix}, S_s)$

$\begin{bmatrix}\alpha_k\\ \beta_k\end{bmatrix} \sim \text{MVNormal}(\begin{bmatrix}0\\0\end{bmatrix}, S_k)$

$\begin{bmatrix}\alpha_y\\ \beta_y\end{bmatrix} \sim \text{MVNormal}(\begin{bmatrix}0\\0\end{bmatrix}, S_y)$

$\begin{bmatrix}\alpha_j\\ \beta_j\end{bmatrix} \sim \text{MVNormal}(\begin{bmatrix}0\\0\end{bmatrix}, S_j)$

$$S_s = \begin{pmatrix} \sigma_{\alpha s}^2 & \sigma_{\alpha s}\sigma_{\beta s}\rho_s \\ \sigma_{\alpha s}\sigma_{\beta s}\rho_s & \sigma_{\beta s}^2 \end{pmatrix}$$
$$S_k = \begin{pmatrix} \sigma_{\alpha k}^2 & \sigma_{\alpha k}\sigma_{\beta k}\rho_k \\ \sigma_{\alpha k}\sigma_{\beta k}\rho_k & \sigma_{\beta k}^2 \end{pmatrix}$$
$$S_y = \begin{pmatrix} \sigma_{\alpha y}^2 & \sigma_{\alpha y}\sigma_{\beta y}\rho_y \\ \sigma_{\alpha y}\sigma_{\beta y}\rho_y & \sigma_{\beta y}^2 \end{pmatrix}$$
$$S_j = \begin{pmatrix} \sigma_{\alpha j}^2 & \sigma_{\alpha j}\sigma_{\beta j}\rho_j \\ \sigma_{\alpha j}\sigma_{\beta j}\rho_j & \sigma_{\beta j}^2 \end{pmatrix}$$
$R_s \sim \text{LKJcorr}(1)$
$R_k \sim \text{LKJcorr}(1)$
$R_y \sim \text{LKJcorr}(1)$
$R_j \sim \text{LKJcorr}(1)$

Interaction models:
$\mu_r \sim \text{Normal}\big(\alpha + \alpha_{s[r]} + \alpha_{k[r]} + \alpha_{y[r]} + \alpha_{j[r]} + \beta_1 * Trt_r + \beta_2 * St_{r,s,k,y,j} + \beta_3 * Trt_r \text{x}\, St_{r,s,k,y,j} + (\beta_{s[r]} + \beta_{k[r]} + \beta_{y[r]} + \beta_{j[r]}) * Trt_r,\ \sigma_r + \sigma_{\bar{y}r}\big)$ eq. 3
$\alpha \sim \text{uniform}\ (-\infty, \infty)$
$\beta_1 \sim \text{uniform}\ (-\infty, \infty)$
$\beta_2 \sim \text{uniform}\ (-\infty, \infty)$
$\beta_3 \sim \text{uniform}\ (-\infty, \infty)$
$\sigma_r \sim \text{student\_t}(3,0,10)$
$\sigma_{\alpha s} = \text{student\_t}(3,0,10)$
$\sigma_{\beta s} = \text{student\_t}(3,0,10)$
$\sigma_{\alpha k} = \text{student\_t}(3,0,10)$
$\sigma_{\beta k} = \text{student\_t}(3,0,10)$
$\sigma_{\alpha y} = \text{student\_t}(3,0,10)$
$\sigma_{\beta y} = \text{student\_t}(3,0,10)$
$\sigma_{\alpha j} = \text{student\_t}(3,0,10)$
$\sigma_{\beta j} = \text{student\_t}(3,0,10)$
$\begin{bmatrix} \alpha_s \\ \beta_s \end{bmatrix} \sim \text{MVNormal}(\begin{bmatrix} 0 \\ 0 \end{bmatrix}, S_s)$
$\begin{bmatrix} \alpha_k \\ \beta_k \end{bmatrix} \sim \text{MVNormal}(\begin{bmatrix} 0 \\ 0 \end{bmatrix}, S_k)$
$\begin{bmatrix} \alpha_y \\ \beta_y \end{bmatrix} \sim \text{MVNormal}(\begin{bmatrix} 0 \\ 0 \end{bmatrix}, S_y)$
$\begin{bmatrix} \alpha_j \\ \beta_j \end{bmatrix} \sim \text{MVNormal}(\begin{bmatrix} 0 \\ 0 \end{bmatrix}, S_j)$
$$S_s = \begin{pmatrix} \sigma_{\alpha s}^2 & \sigma_{\alpha s}\sigma_{\beta s}\rho_s \\ \sigma_{\alpha s}\sigma_{\beta s}\rho_s & \sigma_{\beta s}^2 \end{pmatrix}$$
$$S_k = \begin{pmatrix} \sigma_{\alpha k}^2 & \sigma_{\alpha k}\sigma_{\beta k}\rho_k \\ \sigma_{\alpha k}\sigma_{\beta k}\rho_k & \sigma_{\beta k}^2 \end{pmatrix}$$
$$S_y = \begin{pmatrix} \sigma_{\alpha y}^2 & \sigma_{\alpha y}\sigma_{\beta y}\rho_y \\ \sigma_{\alpha y}\sigma_{\beta y}\rho_y & \sigma_{\beta y}^2 \end{pmatrix}$$
$$S_j = \begin{pmatrix} \sigma_{\alpha j}^2 & \sigma_{\alpha j}\sigma_{\beta j}\rho_j \\ \sigma_{\alpha j}\sigma_{\beta j}\rho_j & \sigma_{\beta j}^2 \end{pmatrix}$$
$R_s \sim \text{LKJcorr}(1)$
$R_k \sim \text{LKJcorr}(1)$
$R_y \sim \text{LKJcorr}(1)$
$R_j \sim \text{LKJcorr}(1)$

Supplementary Methods 2. **Site-specific phenology definitions.** Information for each species included in this analysis and whether phenology measurements are recorded at the plot or individual plant level.

https://github.com/cour10eygrace/OTC_synthesis_analyses/blob/master/Supplemental_materials/Methods2.csv

References

1. Bjorkman, A. D., Elmendorf, S. C., Beamish, A. L., Vellend, M. & Henry, G. H. R. Contrasting effects of warming and increased snowfall on Arctic tundra plant phenology over the past two decades. *Glob. Chang. Biol.* **21**, 4651–4661 (2015).
2. Gillespie, M. A. K., Baggesen, N. & Cooper, E. J. High Arctic flowering phenology and plant-pollinator interactions in response to delayed snow melt and simulated warming. *Environ. Res. Lett.* **11**, (2016).
3. Hollister, R. D., Webber, P. J., Nelson, F. E. & Tweedie, C. E. Soil thaw and temperature response to air warming varies by plant community: Results from an open-top chamber experiment in northern Alaska. *Arctic, Antarct. Alp. Res.* **38**, 206–215 (2006).
4. Oberbauer, S. F. *et al.* Tundra CO2 fluxes in response to experimental warming across latitudinal and moisture gradients. *Ecol. Monogr.* **77**, 221–238 (2007).
5. Darrouzet-Nardi, A. *et al.* Limited effects of early snowmelt on plants, decomposers, and soil nutrients in Arctic tundra soils. *Ecol. Evol.* **9**, 1820–1844 (2019).
6. Molau, U. & Alatato, J. M. Responses of subarctic-alpine plant communities to simulated environmental change: Biodiversity of bryophytes, lichens, and vascular plants. *Ambio* **27**, 322–329 (1998).
7. Pedersen, C. & Post, E. Interactions between herbivory and warming in aboveground biomass production of arctic vegetation. *BMC Ecol.* **8**, 1–12 (2008).
8. Natali, S. M., Schuur, E. A. G. & Rubin, R. L. Increased plant productivity in Alaskan tundra as a result of experimental warming of soil and permafrost. *J. Ecol.* **100**, 488–498 (2012).
9. Fosaa, A. M. The relative contribution of grazing and climate variation on vegetation change in alpine area. *Fróðskaparrit - Faroese Sci. J.* **62**, (2015).
10. Totland, Ø. & Alatalo, J. M. Effects of temperature and date of snowmelt on growth, reproduction, and flowering phenology in the arctic/alpine herb, Ranunculus glacialis. *Oecologia* **133**, 168–175 (2002).
11. Carbognani, M., Tomaselli, M. & Petraglia, A. Different temperature perception in high-elevation plants: new insight into phenological development and implications for climate change in the alpine tundra. *Oikos* **127**, 1014–1023 (2018).
12. Smith, J., Sconiers, W., Spasojevic, M., Ashton, I. & Suding, K. Phenological changes in alpine plants in response to increased snowpack, temperature, and nitrogen. *Arctic, Antarct. Alp. Res.* **44**, 135–142 (2012).
13. Kopp, C. W. & Cleland, E. E. A range-expanding shrub species alters plant phenological response to experimental warming. *PLoS One* **10**, 9–11 (2015).
14. Makowski, D., Ben-Shachar, M. & Lüdecke, D. bayestestR: Describing Effects and their Uncertainty, Existence and Significance within the Bayesian Framework. *J. Open Source Softw.* **4**, 1541 (2019).
15. Bürkner, P.-C. brms : An R package for bayesian multilevel models using Stan. *J. Stat. Softw.* **80**, (2017).
16. Kruschke, J. K. *Doing Bayesian Data Analysis: A Tutorial with R and BUGS*. (Academic Press, Inc., 2010).
17. Wickham, H. *Elegant Graphics for Data Analysis*. *Media* **35**, (Springer Publishing Company, Incorporated, 2009).
18. Therneau, T. A package for survival analysis in S. *Citeseer* 1–83 (2020).
19. van de Pol, M. & Wright, J. A simple method for distinguishing within- versus between-subject effects using mixed models. *Anim. Behav.* **77**, 753–758 (2009).
