# Supplementary material for: Experimental warming differentially affects vegetative and reproductive phenology of tundra plants
Source: Nat Commun. 2021 Jun 11;12:3442. doi: 10.1038/s41467-021-23841-2 (PMC8196023; doi:10.1038/s41467-021-23841-2)
Supplement: Supplementary file 2 — Reporting Summary [file 41467_2021_23841_MOESM2_ESM.pdf]

nature research

Corresponding author(s): Courtney G. Collins

Last updated by author(s): May 13, 2021

Reporting Summary

Nature Research wishes to improve the reproducibility of the work that we publish. This form provides structure for consistency and transparency in reporting. For further information on Nature Research policies, see our Editorial Policies and the Editorial Policy Checklist.

Statistics

For all statistical analyses, confirm that the following items are present in the figure legend, table legend, main text, or Methods section.

n/a	Confirmed	
☐	☒	The exact sample size (n) for each experimental group/condition, given as a discrete number and unit of measurement
☐	☒	A statement on whether measurements were taken from distinct samples or whether the same sample was measured repeatedly
☒	☐	The statistical test(s) used AND whether they are one- or two-sided<br>*Only common tests should be described solely by name; describe more complex techniques in the Methods section.*
☐	☒	A description of all covariates tested
☐	☒	A description of any assumptions or corrections, such as tests of normality and adjustment for multiple comparisons
☐	☒	A full description of the statistical parameters including central tendency (e.g. means) or other basic estimates (e.g. regression coefficient) AND variation (e.g. standard deviation) or associated estimates of uncertainty (e.g. confidence intervals)
☒	☐	For null hypothesis testing, the test statistic (e.g. F, t, r) with confidence intervals, effect sizes, degrees of freedom and P value noted<br>*Give P values as exact values whenever suitable.*
☐	☒	For Bayesian analysis, information on the choice of priors and Markov chain Monte Carlo settings
☐	☒	For hierarchical and complex designs, identification of the appropriate level for tests and full reporting of outcomes
☐	☒	Estimates of effect sizes (e.g. Cohen's d, Pearson's r), indicating how they were calculated

Our web collection on statistics for biologists contains articles on many of the points above.

Software and code

Policy information about availability of computer code

Data collection	We used the statistical program R (v 3.6.1) and the R package 'Taxonstand' (v 2.3) to standardize plant species names in our dataset
Data analysis	We used the statistical program R (v 3.6.1), and the program Stan accessed through the R package 'brms' (v 2.14.4) as well as the 'survival' (v 3.2.7) and 'BayestestR' (v 0.9.0) packages to run all statistical analyses. All analysis code can be found at https://github.com/cour10eygrace/OTC_synthesis_analyses.git.

For manuscripts utilizing custom algorithms or software that are central to the research but not yet described in published literature, software must be made available to editors and reviewers. We strongly encourage code deposition in a community repository (e.g. GitHub). See the Nature Research guidelines for submitting code & software for further information.

Data

Policy information about availability of data

All manuscripts must include a data availability statement. This statement should provide the following information, where applicable:

- Accession codes, unique identifiers, or web links for publicly available datasets
- A list of figures that have associated raw data
- A description of any restrictions on data availability

All data used in this analysis and the supplementary appendices can be found at https://github.com/cour10eygrace/OTC_synthesis_analyses.git. Figures 3, 4, S1, and S2 of this manuscript have associated raw data. The complete dataset is also archived in the Polar data Catalogue (https://doi.org/10.21963/13215).

Field-specific reporting

Please select the one below that is the best fit for your research. If you are not sure, read the appropriate sections before making your selection.

☐ Life sciences ☐ Behavioural & social sciences ☒ Ecological, evolutionary & environmental sciences

For a reference copy of the document with all sections, see nature.com/documents/nr-reporting-summary-flat.pdf

Ecological, evolutionary & environmental sciences study design

All studies must disclose on these points even when the disclosure is negative.

Study description	This study is a quantitative synthesis of tundra plant phenology responses to long-term climate warming manipulations, based on 46 experiments conducted at 18 sites in alpine and Arctic locations worldwide, with observations of over 118 plant species spanning from 1992-2019 as part of the International Tundra Experiment. We incorporate multiple plant phenophases across the entire growing season (green-up, flowering, end-of-flowering, fruiting, seed-dispersal, and leaf-senescence) to assess how warming differentially impacts reproductive versus vegetative phenology, as well as early versus late season phenology. Experimental warming is carried out at experimental locations within each site using passive open-top warming chambers (OTCs) made of fiberglass in either cone or hexagon shape approximately 1.5-2 m in diameter. We use interval-censored modeling and Bayesian hierarchical modeling to control for sources of variation within the data including: differences in sampling intervals, site location, sub-site location (e.g. experimental location nested within site), plant species identity, and time of sampling (year nested within site). We also examine interactions between experimental warming (i.e. treatment) and the following covariates: 1) Years of warming (continuous, replicate level), 2) latitude (continuous, site level), 3) water availability (categorical:dry/moist/wet) based on gravimetric water content (GWC), site:subsite level), 4) OTC deployment period (categorical (year-round/summer only), site level), 5) site mean temperature (continuous, site level) and 6) site-year temperature anomaly (continuous, site:year level). Number of observations, sites, subsites, years and species for each plant phenophase and number of replicates included in each hierarchical model can be found in Table 2.
Research sample	Research sample includes recorded dates of phenological events (e.g. first flowering date, first leaf color change) for each plant species being monitored at a given site at either the individual plant or plot level. We chose this sample type in order to understand the influence of warming on the timing of plant phenology. Samples are intended to represent a population of each monitored plant species at each experimental location within a site. Phenology observations were taken using a common protocol outlined in the ITEX manual (Molau, U. & MØlgaard, 1996), yet sites included slightly different phenology definitions across sub-sites (experimental plots) and species (e.g. flower open vs. bud break), and we included whichever phenophase definition was most commonly measured at a given sub-site for each species across all years. We then grouped these measurements across all sites and categorized them as one of the six standardized phenophases above. All site and species specific phenology definitions can be found in Appendix S2.
Sampling strategy	Following the ITEX protocol, observers recorded the phenological status of plants one to three times per week over the snow-free season. This frequency allowed for estimation of start and end dates of different phenophases without putting undue burden on researchers (i.e., taking phenology observations every single day would be too time consuming and logistically not feasible at some sites). We used interval censored modeling to control for differences in sampling intervals across sites. Minimum sample size in the field was 20 experimentally warmed individuals (plants) and 20 control plants (across all species) for each monitoring subsite in a given sampling year as indicated in the ITEX manual. In hierarchical models we required at least two measurements of each species in both OTCs and control plots in a given site x subsite x year combination. Rationale for this cutoff follows the advice of Gelman and Hill (2007) that 'even two observations per group is enough to fit a multilevel model' (p 276).
Data collection	Site PIs, Postdocs, graduate students and/or field technicians recorded the phenological status of plants through visual, in person observations one to three times per week over the snow-free season. Measurements were recorded in the field using standardized field data sheets for each species available in the appendix of the ITEX manual (pg IX-XXI).
Timing and spatial scale	Depending on site, data collection started as early as 1992, through as recent as 2019. Years of data collection varied across sites due to differences in funding, and the specific years included at each site can be found in Table 1 and Appendix S3d. Following the ITEX protocol, observers recorded the phenological status of plants one to three times per week over the snow-free season (approximately May 1-September 1). This frequency allowed for estimation of start and end dates of different phenophases without putting undue burden on researchers (i.e., taking phenology observations every single day would be too time consuming and logistically not feasible at some sites). We used interval censored modeling to control for differences in sampling intervals across sites. The spatial scale of this analysis is shown in Fig 2 and the spatial scale of sample collection was the size of open-top warming chambers (OTCs) 1.5-2 m in diameter and uniformly sized control plots (1.5-2m x 1.5-2m).
Data exclusions	All exclusion criteria were established before analyses began. We excluded any species x sub-site x year combination where more than 20% of the total observations were NAs, for green-up and leaf-senescence only, because missing data can bias effect sizes and because these phenophases at the beginning and end of the growing season are particularly prone to missing data, as the phenological event may have already occurred before the first visit date or may have occurred after the last visit date. Prior to regression, we also discarded any spp x subsite x year combinations that did not have at least two observations in both OTC and control treatments and removed outliers where the difference in OTC vs. control was greater than 4 standard deviations from the mean for that phenophase.
Reproducibility	This is a data synthesis, with field monitoring data collected over time, so we can not reproduce the experiments per se. However all all data and code for analyses from this synthesis will be made publicly available so that others may use our analytical approach with their own phenological data.

Randomization	The locations of control and experimentally warmed plots were randomized within each study site. After appropriately sized plots within each sub-site were established, site PIs randomly selected which plot would be assigned to a 'control' or 'warmed' treatment.
Blinding	Blinding was not possible in this study due to requirements of field researchers to know the species being monitored in order to visually identify their phenological stages. Also researchers could not be blinded to experimental treatments as that would require removing OTCs in the field while sampling multiple times a week which would render the treatments ineffectual.

Did the study involve field work? ☒ Yes ☐ No

Field work, collection and transport

Field conditions	Field sites are Arctic and alpine tundra locations with cold harsh winters and short summer growing seasons. Sites had average summer temperatures that ranged between 2.8 to 11.9 degrees C and daily climate data for each individual site can be found at at https://github.com/cour10eygrace/OTC_synthesis_analyses.git. Phenology observations were taken over the summer (~May 1-September 1) at the different sites, starting on the date that plots were snow-free and continuing through leaf senescence (or first persistent snowfall) in autumn.
Location	Please see Table 1 and Table S1 for information on all locations included in this study
Access & import/export	Phenology observations were taken by site PIs, Postdocs, graduate students and field technicians and were recorded on field data sheets and then sent to the lead author electronically, so no export of samples was necessary. The appropriate permits to access research sites were obtained whenever necessary and the permits/permissions necessary varied among the different sites. Specific permitting information providing access to field sites is as follows: Adventdalen: Store Norske Spitsbergen Kullkompani A/S 06/792/051.5/PCF and Longyearbyen Local Styre (2009) 401-2 sak 34/09, Alexandra Fjord: Qikiqtani Inuit Association and Nunavut Dept of Environment (1989), Gavia Pass: Stelvio National Park (2008), Latnjajaure: Abisko Scientific Research Station permission for long-term experiment "Linking plant and soil ecology" (1994), Kangerlussaq:Government of Greenland (2002), Niwot Ridge: University of Colorado Mountain Research Station and Arapaho and Roosevelt National Forests Special Use Permit (1994), White Mountains: Inyo National Forest Special Use Permit (2014), Endalen: Longyearbyen Lokal Styre (2014) 456-2-X70, Toolik Lake: Bureau of Land Management Alaska Northern Field Office (1994), Atqasuk & Utqiagvik: Ukpeagvik Iñupiat Corporation (1994), Healy: Site permissions under LAS-24220 issued to the University of Alaska by the State of Alaska Department of Natural Resources (2004), Jakobshorn & Val Bercla: Swiss Federal Institute for Forest, Snow and Landscape Research WSL (1992), Daring Lake: Wek'eezhii Land and Water Board (2009), Imnavait Creek: Bureau of Land Management Central Yukon Field Office (2009)#FF09S602 , Faroe Islands: Museum of Natural History of the Faroe Islands (2001), Finse: Hallingskarvet National Park Board (2006)
Disturbance	Data collectors only observed phenology of plants in plots, and did not disturb or collect the plants. At many of the tundra sites, wooden walkways were constructed to minimize compaction and trampling when observers are accessing monitoring plots. The experimental warming treatments were accomplished with plastic open-topped chambers that were placed around plants in plots, but this did not involve disturbing any plants, unless plants were growing exactly on the edge of plots where the plastic siding of the open topped chambers was placed.

Reporting for specific materials, systems and methods

We require information from authors about some types of materials, experimental systems and methods used in many studies. Here, indicate whether each material, system or method listed is relevant to your study. If you are not sure if a list item applies to your research, read the appropriate section before selecting a response.

Materials & experimental systems

n/a	Involved in the study	
☒	☐	Antibodies
☒	☐	Eukaryotic cell lines
☒	☐	Palaeontology and archaeology
☒	☐	Animals and other organisms
☒	☐	Human research participants
☒	☐	Clinical data
☒	☐	Dual use research of concern

Methods

n/a	Involved in the study	
☒	☐	ChIP-seq
☒	☐	Flow cytometry
☒	☐	MRI-based neuroimaging
